# Development and Validation of a Computer Vision-Based Artificial Intelligence System (FenoParasite) for the Microscopic Detection of *Ascaris lumbricoides* and *Giardia lamblia* in Stool Samples

**DOI:** 10.3390/tropicalmed11090246

**Published:** 2026-08-31

**Authors:** Miguel Hueda-Zavaleta, Exequiel Federico Espeche, Francisco Zea Gamboa, Mady Canelu Ramos Rojas, Enrique Lanchipa Valencia, Juan Carlos Gómez de la Torre Pretell

**Affiliations:** 1 Facultad de Ciencias de la Salud, Universidad Privada de Tacna, Tacna 23003, Peru; exeespeche@upt.pe (E.F.E.); frazea@upt.pe (F.Z.G.); canelu.ramos.rojas@gmail.com (M.C.R.R.); elanchipa@upt.pe (E.L.V.); 2 Clinical Laboratory Roe, Lima 15076, Peru; jgomez@labroe.com; 3 Facultad de Medicina, Universidad Ricardo Palma, Lima 15039, Peru

**Keywords:** artificial intelligence, computer vision, stool microscopy, *Ascaris lumbricoides*, *Giardia lamblia*, parasitic diseases, diagnostic accuracy

## Abstract

Background: Intestinal parasitic infections caused by *Giardia lamblia* and *Ascaris lumbricoides* remain a public-health problem in resource-limited settings, where conventional microscopy depends on expert personnel and shows variable sensitivity. Artificial intelligence (AI) could standardize and decentralize diagnosis. This study aimed to design and validate FenoParasite, an AI object-detection software (Azure Custom Vision) for the microscopic identification of *G. lamblia* cysts and trophozoites and *A. lumbricoides* eggs in stool samples. Methods: Diagnostic-accuracy study. The reference standard was consensus microscopy by two expert readers, with a third reader for discordant cases. An internal validation set (*n* = 159) and a prospective pilot validation set (*n* = 31) were analyzed. Sensitivity, specificity, positive predictive value, negative predictive value, accuracy (95% Clopper–Pearson CI), AUC-ROC (bootstrap), and Cohen’s kappa were computed; the confidence threshold was 90%. Results: The model achieved a mAP@0.30 of 98.3% (precision 98.2%; recall 96.4%). In internal validation, *G. lamblia* showed 100% sensitivity, 98.9% specificity, 99.4% accuracy, and AUC 0.98; *A. lumbricoides* reached 100% across all indices (AUC 1.00). In the prospective pilot validation, sensitivity and specificity were 100% for both taxa; however, the confidence intervals were very wide (lower sensitivity bounds of 39.8% and 75.3%, based on only 4 and 13 reference-positive specimens, respectively), and these estimates are correspondingly imprecise. Conclusions: FenoParasite showed high agreement with consensus expert microscopy for both a protozoan and a helminth. Because the reference standard was expert microscopy rather than a molecular assay, the reported performance estimates quantify agreement with microscopic reading rather than absolute diagnostic accuracy. These findings remain preliminary. Multicenter validation against molecular reference standards is required before clinical implementation can be considered.

## 1. Introduction

Intestinal parasitic infections remain one of the leading and most persistent causes of morbidity worldwide, particularly in populations with limited access to safe water and adequate sanitation. More than 1.5 billion people (approximately 24% of the global population), predominantly school-aged children between 5 and 14 years of age, are estimated to be infected with soil-transmitted helminths (STHs), among which *A. lumbricoides* is one of the most prevalent species globally [1,2]. In addition, intestinal protozoa, particularly *G. lamblia*, are major causes of persistent diarrhea, malabsorption, malnutrition, and growth impairment in children [3,4]. In Peru, enteroparasitic infections remain an important public health problem, with prevalence varying across communities according to parasite bioecological characteristics [3,5].

Conventional diagnosis relies primarily on direct light microscopy of stool specimens, a widely available and inexpensive technique whose diagnostic performance depends heavily on the expertise of the microscopist, examination time, and specimen quality. Consequently, conventional microscopy is subject to considerable interobserver variability, reduced sensitivity in low-intensity infections, and an increased risk of missed or delayed diagnoses, particularly in laboratories facing workforce shortages or high diagnostic workloads [6]. These limitations underscore the need for technologies capable of standardizing and accelerating parasite identification while preserving the accessibility of routine laboratory workflows.

Artificial intelligence (AI), particularly deep learning based on convolutional neural networks (CNNs) for computer vision, has demonstrated outstanding performance in automated biomedical image analysis, frequently achieving diagnostic accuracy comparable to—or even exceeding—that of experienced human observers [7]. In parasitology, AI algorithms can detect and classify parasitic structures in microscopic images, offering a promising approach to improve diagnostic efficiency, optimize limited laboratory resources, and enhance the reproducibility of microscopic examinations, especially in resource-constrained settings with a high burden of disease [8]. Computer vision-based clinical decision support systems (CDSSs) integrated into digital microscopy workflows may therefore improve the timeliness and consistency of parasitological diagnosis.

Despite this potential, most existing AI applications in intestinal parasitology have focused on a single parasite species or developmental stage and have primarily reported object detection performance metrics, such as precision or mean average precision, without evaluating sample-level diagnostic accuracy against an appropriate reference standard. This represents a major limitation for clinical translation and does not fully comply with the current recommendations for diagnostic accuracy studies (STARD 2015) and AI-based medical imaging research (CLAIM) [9,10].

To address this gap, we designed, developed, and validated FenoParasite, a computer vision-based artificial intelligence system trained on digitized microscopic images to simultaneously detect and classify, in real-time, three epidemiologically important parasitic targets: *A. lumbricoides* eggs, *G. lamblia* cysts, and *G. lamblia* trophozoites. The objective of this study was to design and validate FenoParasite as an AI-assisted tool for the identification of human intestinal parasitic infections.

## 2. Materials and Methods

### 2.1. Study Design

A cross-sectional diagnostic accuracy study was conducted to validate the diagnostic performance of the FenoParasite artificial intelligence (AI) system against a parasitological reference standard. The study comprised two methodologically distinct phases: (1) development and internal validation of the object detection model at the image/annotation level, and (2) validation of the AI system’s sample-level diagnostic accuracy against the reference standard. The study was reported in accordance with the Standards for Reporting of Diagnostic Accuracy Studies (STARD 2015) guidelines and the Checklist for Artificial Intelligence in Medical Imaging (CLAIM); both completed checklists are provided as Supplementary Files S1 and S2. The second phase is designated a prospective pilot validation rather than an external validation, because specimen processing, image acquisition, model development and evaluation were all conducted within the same institution using identical equipment, staining protocols, and operators. It therefore assesses temporal and specimen-level independence, but not independence of setting, instrumentation, or operator; true external validation in independent laboratories remains to be performed.

### 2.2. Study Setting and Period

The study was conducted between May 2025 and March 2026. Stool sample collection and laboratory processing were performed at the Microbiology Laboratory of the Universidad Privada de Tacna, while image processing, model training, and validation were carried out in the Microbiology, Microscopy, and Computing Laboratories of the Faculty of Health Sciences, Universidad Privada de Tacna.

### 2.3. Study Population, Samples, and Sampling

The study population consisted of stool samples obtained from patients undergoing screening for intestinal parasitic infections. The unit of analysis was the stool specimen together with its corresponding data collection form. A non-probability convenience sampling strategy was used, including all eligible samples collected during the study period.

Eligible specimens included stool samples from patients with suspected intestinal parasitic infections, regardless of previous parasitological findings. Samples from individuals who had received antiparasitic treatment within the preceding 2–4 weeks, as well as insufficient, poorly preserved, or macroscopically inadequate specimens, were excluded. No formal a priori sample-size calculation was performed. The sample size was determined pragmatically by the number of eligible specimens available during the predefined study period, using a non-probability convenience sampling strategy. The prospective validation phase was intended as a pilot assessment, and its statistical precision was therefore characterized using exact 95% confidence intervals.

### 2.4. Reference Standard

The two reference microscopists (F.Z.G. and M.C.R.R.) examined the original glass slides directly under the microscope, performing a complete systematic sweep of the coverslip area. Thus, the reference standard reflected an examination of the entire preparation, whereas FenoParasite analyzed the microscopic fields visualized during the live validation sweep. This asymmetry could result in apparent false-negative AI classifications when parasites are present outside the fields examined during the real-time AI assessment, potentially leading to an underestimation of model sensitivity.

### 2.5. Image Acquisition and Digitization

Microscopic preparations were examined using an Olympus CX33 trinocular light microscope (Olympus Corporation, Tokyo, Japan), equipped with an Olympus LC35 3.5-megapixel CMOS digital camera and a U-TV0.5XC-3 0.5× camera adapter (Olympus Corporation, Tokyo, Japan). Images were acquired using LCmicro version 2.4 (Evident Corporation, Tokyo, Japan). During specimen-level validation, slides were examined using a systematic zigzag sweep, with approximately five microscopic fields initially assessed at 10× magnification and additional examination at 40× when required. FenoParasite version 1.0. processed the live microscope stream while the slide was moved; therefore, the number of frames analyzed varied between specimens and individual frames were not routinely archived. The operator performing image acquisition was blinded to the reference microscopy result.

### 2.6. Dataset Construction and Image Annotation

The dataset consisted of high-resolution digitized microscopic images. Image annotation was performed manually by specialists in medical parasitology, who delineated each parasite structure using bounding boxes, ensuring accurate taxonomic and morphological labeling. The annotated dataset comprised 148 annotations of *A. lumbricoides* eggs, 71 annotations of *G. lamblia* cysts, and 95 annotations of *G. lamblia* trophozoites.

### 2.7. AI Model Development and Training

Real-time detection and classification of the three parasite categories were implemented using a convolutional neural network model developed with Microsoft Azure Custom Vision (Microsoft Corporation, Redmond, WA, USA; web-based platform, version not applicable; available online: https://www.customvision.ai/; accessed on 23 March 2026). An object detection architecture optimized for high-performance, low-latency inference was selected to facilitate integration into computer-assisted microscopy workflows. Model training was performed using the platform’s Advanced Training mode on cloud-based GPU clusters, requiring approximately 1–3 h of dedicated computation time. To improve model robustness against variations in microscopic image acquisition, native data augmentation techniques—including rotation, translation, brightness adjustment, contrast modification, and scaling—were applied.

### 2.8. Dataset Partitioning and Internal Model Validation

Internal object-detection performance was evaluated using the automated hold-out procedure implemented by Azure Custom Vision. Partitioning was performed at the individual-image level rather than at the specimen level; therefore, different images originating from the same calibration specimen could potentially have been allocated to both the training and evaluation subsets. This introduces a potential source of data leakage, and the resulting precision, recall, AP, and mAP values should therefore be interpreted as internal model-development metrics rather than independent estimates of generalization performance. Importantly, the 159 stool specimens subsequently used for specimen-level diagnostic validation were independent of the image bank used for model training and were not included in the Azure training dataset. Model performance at the bounding-box level was evaluated using the platform-default 80% probability threshold and a 30% intersection-over-union (IoU) threshold; the resulting metrics are reported as AP@0.30 and mAP@0.30. The archived Azure project records did not retain a reliable class-specific breakdown of the number of images and annotated objects assigned to each internal partition; these counts therefore could not be reconstructed retrospectively.

### 2.9. Index Test, Operating Threshold, and Clinical Decision Workflow

The index test consisted of the binary classification generated by FenoParasite (positive/negative). For the real-time diagnostic module integrated into the application, a strict filtering strategy was implemented whereby only detections with a confidence score of ≥90% were automatically accepted and reported. This operating threshold was established during application development, before the specimen-level validation analyses, and was not modified during validation. It was selected to minimize false-positive detections caused by common microscopic artifacts—including cellular debris, air bubbles, starch granules, and plant fibers—that may resemble parasitic structures. In addition, the threshold was intended to maximize agreement with expert microscopists and improve specificity while reducing the impact of image variability arising from illumination changes or streaming artifacts during real-time image acquisition.

Two distinct thresholds are reported in this study and should not be conflated. The 80% probability threshold is the default applied by the Azure Custom Vision platform when computing object-detection metrics at the level of individual bounding boxes, and is a property of the platform evaluation routine. The 90% confidence threshold is the value required for a detection to be accepted by the FenoParasite application in the specimen-level diagnostic workflow, and it underlies all specimen-level indices reported in this article.

Specimen-level decision rule. Each specimen contributed multiple microscopic fields, and each field could contain zero, one, or several detected objects. Aggregation from object level to specimen level followed a prespecified any-positive rule: for a given target class (*A. lumbricoides* egg, *G. lamblia* cyst, or *G. lamblia* trophozoite), a specimen was classified as positive for that class if at least one detected object of that class with a confidence score of 90% or higher was present in at least one microscopic field analyzed during the live validation sweep. No minimum object count per field or per specimen was required. The consolidated specimen-level result for *G. lamblia* was positive if the specimen was positive for cysts, for trophozoites, or for both. Because a specimen can be classified as positive on the strength of a single qualifying detection among many fields, this rule increases the specimen-level sensitivity relative to per-field sensitivity while reducing specimen-level specificity, since each additional field represents an additional opportunity for a false-positive detection.

### 2.10. Study Variables

The primary variables included:

▪ Index test: FenoParasite classification for *A. lumbricoides*, *G. lamblia* cysts, *G. lamblia* trophozoites, and consolidated *Giardia* detection, together with the associated prediction confidence score (%).
▪ Reference standard: expert microscopy diagnosis (positive/negative for each target parasite).
▪ Patient and specimen characteristics: age, age group, sex, area of residence (urban, peri-urban, or rural), macroscopic specimen quality, *Ascaris* egg burden (eggs per microscopic field and parasite density category), and the presence of other intestinal parasites.
▪ Technical imaging variables: microscope, digital camera, acquisition software, magnification, number of images, image resolution, image format, image quality, model iteration, and validation dataset (internal evaluation or prospective pilot validation).
▪ Diagnostic agreement: specimen-level results for *A. lumbricoides* and consolidated *G. lamblia* detection were classified as true positive, true negative, false positive, or false negative relative to the reference standard.

### 2.11. Statistical Analysis

Descriptive analyses were performed for demographic, clinical, and technical variables. Categorical variables were summarized as frequencies and percentages, whereas continuous variables were reported as mean ± standard deviation (SD) or median with interquartile range (IQR), according to their distribution. Specimen-level diagnostic performance was evaluated by constructing confusion matrices for *A. lumbricoides* and the consolidated *G. lamblia* endpoint. Sensitivity, specificity, positive predictive value (PPV), negative predictive value (NPV), likelihood ratios, and overall diagnostic accuracy were calculated with corresponding 95% confidence intervals. Discriminative performance was assessed using the area under the receiver operating characteristic curve (AUC-ROC) based on the prediction probabilities generated by the AI model. Agreement between FenoParasite and the reference standard, as well as interobserver agreement between microscopists, was evaluated using Cohen’s kappa coefficient (κ). Object detection performance was reported as the precision, recall, average precision (AP) for each parasite class, and mean average precision (mAP) across all classes. Statistical analyses were conducted using Microsoft Excel for Microsoft 365, version 2607 (Microsoft Corporation, Redmond, WA, USA), and Stata version 17 (StataCorp LLC, College Station, TX, USA), with a two-sided *p*-value < 0.05 considered statistically significant. For the receiver operating characteristic analysis, a single continuous specimen-level score was derived for each target using maximum pooling, defined as the highest raw confidence score returned by the model across all microscopic fields analyzed during the live validation sweep for that specimen, before application of the predefined 90% clinical operating threshold. Raw confidence scores below 0.90 were retained for this analysis, and a score of zero was assigned only when the model generated no detection for the target class. The AUC was calculated from these continuous specimen-level scores against the binary reference standard. The predefined 90% threshold was used only for the final specimen-level positive/negative classification and did not restrict the confidence scores used to construct the ROC curve. Finally, because the reference standard was expert light microscopy rather than a molecular assay, the indices reported here quantify agreement with expert microscopic reading rather than absolute diagnostic accuracy against a biological reference standard. No missing index-test or reference-standard results were present among the specimens included in the diagnostic accuracy analyses; therefore, no imputation of diagnostic outcomes was performed.

### 2.12. Ethical Considerations

The study was conducted in accordance with the principles of the Declaration of Helsinki and the International Committee of Medical Journal Editors (ICMJE) recommendations. Ethical approval was obtained from the Research Ethics Committee of the Faculty of Health Sciences, Universidad Privada de Tacna (approval No. FACSA-CEI/058-04-2025; 29 April 2025). Written informed consent was obtained from all adult participants. For pediatric participants, written informed consent was obtained from their parents or legal guardians, and written assent was obtained from children capable of providing assent according to their age and level of understanding.

All data were anonymized to ensure participant confidentiality. The study also complied with applicable policies regarding data and code availability to promote research transparency and reproducibility.

## 3. Results

### 3.1. Sample Flow and Population Characteristics

Two independent datasets were analyzed according to the predefined two-phase study design. The internal sample-level validation included 159 stool samples processed and classified against the reference standard. Expert microscopy confirmed a parasite prevalence of 15.7% (25/159) for *A. lumbricoides* and 41.5% (66/159) for *G. lamblia* (combined cyst and trophozoite forms).

The prospective pilot validation of diagnostic accuracy included 31 prospectively collected stool samples obtained from participants who provided informed consent.

In the prospective pilot validation cohort, the mean age was 11.2 years (SD, 10.7; median, 8 years), with a predominance of pediatric participants. Overall, 58.1% (18/31) were male and 41.9% (13/31) were female, and most participants resided in urban areas (96.8%; 30/31). Regarding macroscopic specimen quality, 74.2% (23/31) of samples were classified as adequate, 19.4% (6/31) as borderline, and 6.5% (2/31) as inadequate. According to the reference standard, parasite prevalence in the prospective pilot validation was 12.9% (4/31) for *A. lumbricoides* and 41.9% (13/31) for *G. lamblia*. The characteristics of both datasets are summarized in Table 1.

### 3.2. Internal Object-Detection Model Evaluation

During the internal annotation-level validation, FenoParasite showed high object-detection performance, achieving an overall precision of 98.2%, recall of 96.4%, and mean average precision of 98.3% at an IoU threshold of 0.30 (mAP@0.30). Taxon-specific average precision at the same overlap threshold (AP@0.30) was 97.8% for *A. lumbricoides* eggs (148 annotations), 97.6% for *G. lamblia* cysts (71 annotations), and 99.4% for *G. lamblia* trophozoites (95 annotations) (Table 2).

### 3.3. Sample-Level Diagnostic Accuracy: Internal Validation

**Figure 1:**
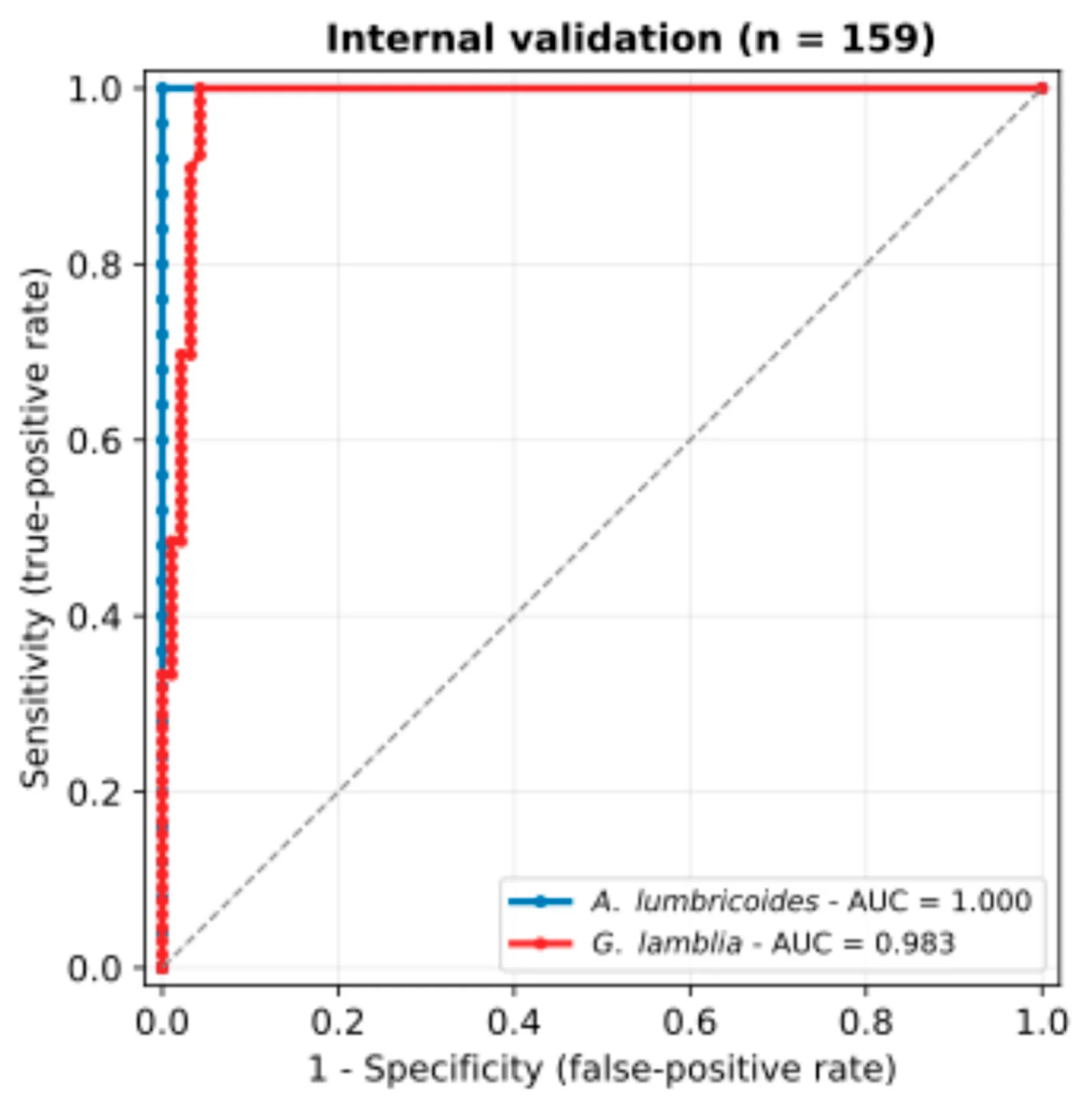
ROC curves of FenoParasite against the reference standard. Receiver operating characteristic (ROC) curves for the detection of *A. lumbricoides* (blue) and *G. lamblia* (red) by FenoParasite, using the model confidence score as the discrimination variable, compared with the expert-microscopy reference standard. Internal validation (*n* = 159): area under the curve (AUC) 1.000 for *A. lumbricoides* and 0.983 (95% CI 0.962–0.999) for *G. lamblia*.

Using the predefined clinical confidence threshold (≥90%) for sample-level positive/negative classification, FenoParasite showed high agreement with the reference standard (Table 3, Figure 1).

For *A. lumbricoides*, the AI system correctly identified 25 true-positive and 134 true-negative samples, with no false-positive or false-negative results. Accordingly, sensitivity, specificity, positive predictive value (PPV), negative predictive value (NPV), and overall accuracy were all 100.0% (sensitivity: 95% CI, 86.3–100.0; specificity: 95% CI, 97.3–100.0; PPV: 95% CI, 86.3–100.0; NPV: 95% CI, 97.3–100.0; accuracy: 95% CI, 97.7–100.0). The area under the receiver operating characteristic curve (AUC-ROC) was 1.00 (95% CI, 1.00–1.00), and agreement with the reference standard was perfect (Cohen’s κ = 1.00).

For *G. lamblia*, 66 true-positive, 92 true-negative, and one false-positive result were identified, with no false-negative results. Sensitivity was 100.0% (95% CI, 94.6–100.0), specificity 98.9% (95% CI, 94.2–100.0), PPV 98.5% (95% CI, 92.0–100.0), NPV 100.0% (95% CI, 96.1–100.0), and overall accuracy 99.4% (95% CI, 96.5–100.0). The positive likelihood ratio (LR+) was 93.0 (95% CI, 13.2–653.3), whereas the negative likelihood ratio (LR−) could not be estimated because no false-negative results were observed. The AUC was 0.983 (95% CI, 0.962–0.999), and agreement with the reference standard was almost perfect (Cohen’s κ = 0.99; 95% CI, 0.96–1.00).

### 3.4. Sample-Level Diagnostic Accuracy: Prospective Pilot Validation

During the prospective pilot validation, FenoParasite correctly classified all samples according to the reference standard for both taxa (Table 4).

For *A. lumbricoides*, the AI system correctly identified 4 true-positive and 27 true-negative samples, with no false-positive or false-negative results. Consequently, sensitivity, specificity, positive predictive value (PPV), negative predictive value (NPV), and overall accuracy were all 100.0%. Because of the limited number of positive cases, confidence intervals were relatively wide (sensitivity: 95% CI, 39.8–100.0; specificity: 95% CI, 87.2–100.0; accuracy: 95% CI, 88.8–100.0). The area under the receiver operating characteristic curve (AUC) was 1.00, and agreement with the reference standard was perfect (Cohen’s κ = 1.00).

For *G. lamblia*, 13 true-positive and 18 true-negative samples were correctly identified, with no false-positive or false-negative results. Sensitivity, specificity, and overall accuracy were all 100.0% (sensitivity: 95% CI, 75.3–100.0; specificity: 95% CI, 81.5–100.0; accuracy: 95% CI, 88.8–100.0). The AUC was 1.00, and agreement with the reference standard was also perfect (Cohen’s κ = 1.00).

### 3.5. Interobserver Agreement of the Reference Standard

Interobserver agreement between the two independent microscopists who established the reference standard ranged from substantial to almost perfect. During the internal validation, Cohen’s κ was 1.00 for *A. lumbricoides*, 0.88 (95% CI, 0.81–0.96) for *G. lamblia* cysts, and 0.95 (95% CI, 0.86–1.00) for *G. lamblia* trophozoites. A total of 25 discrepant readings (15.7%; 25/159) were identified and resolved by a third expert microscopist according to the predefined consensus protocol.

In the prospective pilot validation, Cohen’s κ was 0.84 (95% CI, 0.53–1.00) for *A. lumbricoides*, 1.00 for *G. lamblia* cysts, and 0.65 (95% CI, wide due to the small number of events) for *G. lamblia* trophozoites. Only one discrepant reading (3.2%; 1/31) was observed and subsequently resolved by expert consensus (Table 5). Intra-rater repeatability was not assessed because each reference microscopist performed a single independent reading of each specimen; inter-rater variability and disagreement resolution were evaluated as described above.

### 3.6. Discordant Results Analysis

**Figure 2:**
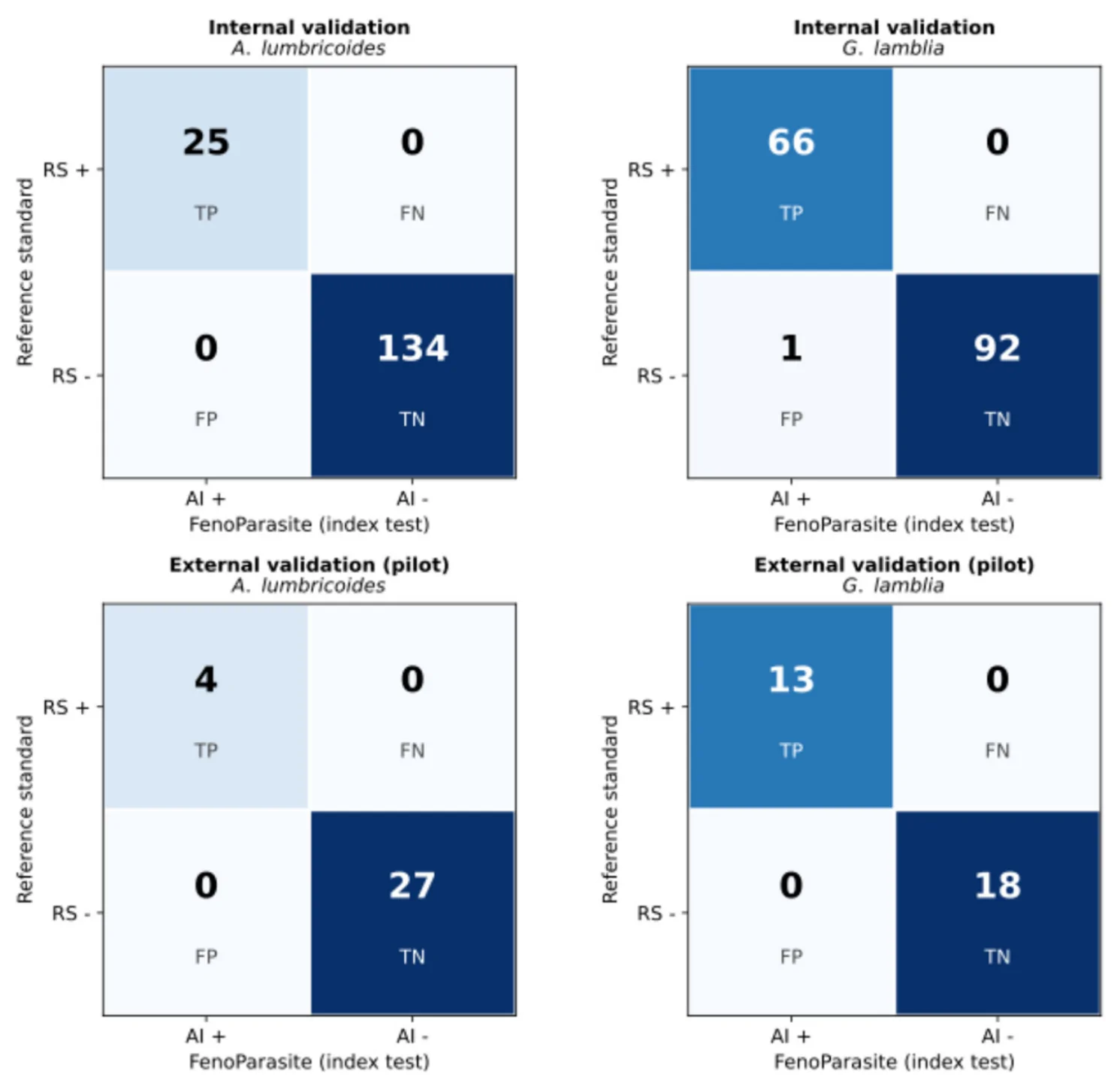
Confusion matrices of FenoParasite against the reference standard. 2 × 2 cross-tabulations of the FenoParasite index test (AI +/−) versus the expert-microscopy reference standard (RS +/−) for *A. lumbricoides* and *G. lamblia* in the internal validation set (top row, *n* = 159) and the prospective pilot validation set (bottom row, *n* = 31). TP, true positive; FN, false negative; FP, false positive; TN, true negative. Cell color intensity represents the number of specimens, with darker blue indicating higher counts. A single false-positive result for *G. lamblia* was observed in the internal validation; no false negatives were recorded in either phase.

Only one discordant result was identified between FenoParasite and the reference standard across the entire study dataset. This corresponded to a false-positive result for *G. lamblia* during the internal validation, in which a sample classified as negative by blinded expert microscopy was identified as positive by the AI system. No false-negative results were observed for either taxon during either the internal evaluation or the prospective pilot validation, corresponding to an observed sensitivity of 100% under the evaluated conditions. This absence of false negatives should not be attributed to the 90% operating confidence threshold: raising a confidence threshold moves the operating point toward higher specificity at the expense of sensitivity, and would if anything be expected to generate additional false negatives at the object level. Three other mechanisms provide a more plausible explanation. First, the specimen-level any-positive aggregation rule required only a single qualifying detection among the multiple fields captured per specimen, so that object-level misses were largely recovered at the specimen level. Second, parasites not visualized by the reference microscopists could not by definition be scored as false negatives because they were absent from the reference standard itself. Third, with 25 and 66 reference-positive specimens for *A. lumbricoides* and *G. lamblia*, respectively, the upper confidence bounds on the false-negative rate remain compatible with materially lower true sensitivities; the observation of zero false negatives must therefore not be read as evidence of perfect detection (Figure 2).

## 4. Discussion

In this study, we developed and validated FenoParasite, a computer vision-based artificial intelligence system for the automated identification of *G. lamblia* (cysts and trophozoites) and *A. lumbricoides* eggs in direct Lugol-stained stool microscopy. The system showed high performance in both object detection (mean average precision at an IoU threshold of 0.30 [mAP@0.30] of 98.3%) and sample-level agreement with the reference standard. During internal validation, FenoParasite achieved 100% sensitivity for both parasites, with specificities of 100% for *A. lumbricoides* and 98.9% for *G. lamblia*. In the prospective pilot validation, all specimens were classified concordantly with the reference standard for both taxa, although with wide confidence intervals. Notably, no false-negative results were observed in any validation phase, and only a single false-positive result was identified for *G. lamblia* across the entire dataset, although, as discussed below, these estimates are constrained by the use of an imperfect reference standard and by the limited size of the validation cohorts, and should be regarded as preliminary.

### 4.1. Comparison with Previous Studies

FenoParasite showed high performance at both the object-detection and specimen-level stages. Previous studies have achieved object-level accuracies ranging from 93% to 95%, including Ward et al. (2022) [11], who reported a weighted precision of 94.9% and recall of 96.1% using an R-FCN ResNet101 model on Kato–Katz smears from four endemic countries [11], Butploy et al. (2021), who achieved 93.3% classification accuracy with a custom CNN [12], and Martínez Pastor et al. (2024), who reported test accuracies of up to 93.7% using conventional image processing combined with convolutional neural networks [13]. Although direct comparisons should be interpreted cautiously because of differences in datasets, object definitions, and evaluation metrics, FenoParasite achieved a mean average precision of 98.3% at an IoU threshold of 0.30. It should be emphasized that this overlap criterion is more permissive than the 0.50 threshold conventionally applied in the studies cited above, so these values are not directly comparable and no claim of superiority over previously published systems can be made on this basis.

A major strength of this study is the evaluation of diagnostic accuracy at the sample level, rather than relying exclusively on object-level performance metrics. Many previous studies have reported precision, recall, F1-score, or mAP without translating these results into clinically meaningful diagnostic indicators. Among studies that performed clinical validation, diagnostic performance has been variable. Lundin et al. (2024) reported 80% sensitivity and 98.2% specificity for *A. lumbricoides* in Kenya [14], Cure-Bolt et al. (2024) reported 77% sensitivity for an AI-assisted system in the Peruvian Amazon [15], and von Bahr et al. (2025) demonstrated that expert-assisted AI achieved substantially higher sensitivity than either autonomous AI or conventional microscopy [16]. In comparison, FenoParasite achieved 100% sensitivity and 98.9–100% specificity for both *A. lumbricoides* and *G. lamblia*, with no false-negative results during either the internal evaluation or the prospective pilot validation. Nevertheless, these findings should be interpreted cautiously given the limited sample size and spectrum of cases included in the validation cohorts, and given that the reference standard in the present study was expert microscopy rather than the molecular or composite standards used in some of the comparisons cited above.

### 4.2. Detection of G. lamblia and Diagnostic Reliability

Most AI applications in intestinal parasitology have focused on helminth eggs because of their larger size and distinctive morphology [11,14,15,17], whereas protozoa such as *G. lamblia* remain considerably more challenging owing to the small size of cysts and trophozoites and their resemblance to microscopic artifacts. Only a few previous studies have addressed automated *Giardia* detection, primarily using permanent stains or fluorescence microscopy under controlled laboratory conditions [18,19]. In contrast, FenoParasite achieved 100% sensitivity and 98.9% specificity for *G. lamblia* using direct Lugol-stained bright-field stool microscopy, without the need for permanent staining or fluorescence. This suggests potential applicability in routine laboratory workflows, provided that the implementation requirements set out in Section 4.4 can be met.

A key design feature was the use of a 90% confidence threshold, which was intended to minimize false-positive detections arising from morphologically ambiguous artifacts. Previous AI-based systems have reported substantial false-positive rates or relatively low positive predictive values due to confusion with biological artifacts [11,14,17]. In comparison, FenoParasite produced only one false-positive result across all evaluated samples and no false-negative results during either the internal evaluation or the prospective pilot validation. The apparent balance between high specificity and high sensitivity is relevant for screening applications, where missing true infections has greater clinical consequences than occasional false-positive findings. As noted above, however, the observed sensitivity represents an upper bound rather than an established value, because it was estimated against a reference standard that shares the same morphological basis as the index test.

### 4.3. Clinical Implications, Strengths, and Limitations

A major advantage of AI-assisted stool microscopy is its ability to detect low-intensity infections that may be missed by conventional microscopy. Previous studies have shown improved detection of low parasite burdens using AI-based systems, particularly for *A. lumbricoides* and *G. lamblia* [14,15,16,18]. The absence of false-negative results in both the internal evaluation and the prospective pilot validation of FenoParasite is compatible with these findings and suggests potential value as a decision-support tool for screening programs and routine laboratory diagnosis. It must be emphasized, however, that the present design cannot demonstrate the detection of infections missed by conventional microscopy, because organisms not visualized by the reference readers were not recorded as false negatives; establishing added sensitivity over conventional microscopy would require a molecular reference standard.

This study has several strengths, including adherence to the STARD 2015 and CLAIM reporting guidelines, the use of a robust reference standard based on blinded double expert reading with consensus resolution of discrepancies, the simultaneous detection of both a protozoan and a helminth (multiclass capability), and most importantly, the evaluation of diagnostic accuracy at the sample level in addition to object-detection performance, through both an internal evaluation and a prospective pilot validation.

Several limitations should also be acknowledged. First, the prospective pilot validation was conducted with a relatively small sample size (*n* = 31) and a limited number of positive cases, resulting in wide confidence intervals (e.g., 100% sensitivity for *A. lumbricoides*; 95% CI, 39.8–100.0). Therefore, these estimates should be considered preliminary rather than definitive evidence of perfect diagnostic performance. Second, this was a single-center study conducted predominantly in an urban population, which may limit the generalizability of the findings and does not capture the preanalytical variability encountered in rural or multicenter settings. Third, Azure Custom Vision used an image-level hold-out rather than a specimen-level split, creating a potential risk of data leakage and optimistic object-detection metrics. This limitation does not affect the specimen-level diagnostic validation, because the 159 validation specimens were independent of the training image bank. Fourth, conventional light microscopy served as the reference standard, and despite blinded double reading, a single direct Lugol-stained smear has substantially lower sensitivity than molecular methods such as PCR, particularly in low-intensity infections and in the setting of intermittent *Giardia* shedding. Because FenoParasite and the reference readers interpreted the same morphological substrate, their errors are correlated: organisms missed by microscopy would in most instances also be missed by the model and would be classified as true negatives rather than false negatives. This mechanism biases sensitivity and negative predictive value upward, so the values reported here should be regarded as an upper bound, and as a measure of agreement with expert microscopic reading rather than of absolute diagnostic accuracy. Fifth, stage-specific diagnostic performance could not be estimated separately for *G. lamblia* cysts and trophozoites because the prospective validation dataset was structured around a consolidated specimen-level *G. lamblia* endpoint. Although both life stages were treated as separate object classes during model development, complete paired stage-specific reference-standard and AI classifications were not retained for the diagnostic validation analysis. Finally, the cloud-based implementation using Azure Custom Vision raises considerations regarding Internet connectivity and operational costs. Unlike edge-computing approaches described by Ward et al. (2022) [11], these factors should be carefully evaluated before large-scale field deployment.

These findings support the need for large-scale, prospective, multicenter validation across diverse epidemiological settings and parasite burdens, ideally using molecular methods (PCR) as the reference standard to better characterize diagnostic performance in low-intensity infections. Future work should also incorporate parasite burden quantification, expand detection to additional clinically relevant intestinal parasites, evaluate edge-computing implementations to reduce dependence on Internet connectivity, and assess the cost-effectiveness, usability, and real-world clinical impact of FenoParasite in routine laboratory workflows and public health programs.

### 4.4. Implementation Requirements in Resource-Limited Settings

Because applicability in resource-limited settings is a central claim of this work, the practical requirements for deployment should be stated explicitly rather than assumed. FenoParasite requires four components. First, a light microscope with digital image capture; the present implementation used an Olympus CX33 trinocular light microscope equipped with an Olympus LC35 digital camera and a U-TV0.5XC-3 camera adapter, and although the architecture is in principle camera-agnostic and could operate with lower-cost USB eyepiece cameras or smartphone adapters, this compatibility has not yet been formally validated. Second, a computing device with a stable broadband connection, because inference is currently performed through the Azure Custom Vision cloud service; recurrent costs comprise prediction transactions, image storage, and model hosting, and therefore represent a per-specimen marginal cost that must be budgeted before deployment. Third, laboratory consumables for conventional direct examination with Lugol, which already form part of routine practice and add no incremental cost. Fourth, human resources: operating the capture and inference interface does not require expertise in computer science, but a trained microscopist remains necessary to prepare and focus the preparation, to select microscopic fields, and to adjudicate discordant or indeterminate results. FenoParasite is therefore a decision-support tool that standardizes and accelerates microscopic reading rather than a replacement for parasitological expertise, and it does not reduce the requirement for trained laboratory personnel. The dependence on Internet connectivity is the principal barrier to deployment in rural and peri-urban laboratories, where bandwidth is frequently intermittent; migration to an edge-deployable compact model, which the platform permits, would remove both this requirement and the recurrent per-transaction cost, and constitutes a priority for the next development phase.

## 5. Conclusions

FenoParasite showed high agreement with consensus expert microscopy for the identification of *G. lamblia* and *A. lumbricoides* in direct Lugol-stained stool preparations, with an observed sensitivity of 100% and specificity of 98.9–100% at the specimen level. Unlike most previous AI-based studies in intestinal parasitology, this work reports specimen-level indices in addition to object-detection metrics. These findings are nevertheless preliminary and hypothesis-generating: they were obtained in a single center, against an imperfect reference standard that shares the morphological basis of the index test, and with wide confidence intervals in the pilot phase. Confirmation in adequately powered, multicenter studies using molecular or composite reference standards is required before FenoParasite can be considered for clinical implementation.

## Figures and Tables

**Table 1:** Characteristics of the samples analyzed in the internal evaluation and prospective pilot validation phases.

Characteristics	Internal Validation (*n* = 159)	Prospective Pilot Validation (*n* = 31)
Age, mean (SD)	—	11.2 (10.7)
Age, median (range)	—	8 (1–43)
Sex, male, *n* (%)	—	18 (58.1)
Sex, female, *n* (%)	—	13 (41.9)
Urban origin, *n* (%)	—	30 (96.8)
Rural origin, *n* (%)	—	1 (3.2)
Sample quality, adequate, *n* (%)	—	23 (74.2)
Sample quality, borderline, *n* (%)	—	6 (19.4)
Sample quality, inadequate, *n* (%)	—	2 (6.5)
Prevalence of *A. lumbricoides* (GS), *n* (%)	25 (15.7)	4 (12.9)
Prevalence of *G. lamblia* (GS), *n* (%)	66 (41.5)	13 (41.9)

SD: standard deviation; GS: gold standard (optical microscopy by experts with consensus reading).

**Table 2:** Performance metrics of the object detection model by taxon (annotation level, IoU threshold 0.30, probability threshold 80%).

Parasitic Class	Annotations (Bounding Boxes)	Average Precision (AP@0.30)	Average Precision (AP@0.50)
*A. lumbricoides* egg	148	97.8%	
*G. lamblia* cyst	71	97.6%	
*G. lamblia* trophozoite	95	99.4%	
Global (mAP)	314	98.3%	

Overall model precision 98.2%; overall model recall 96.4%. Metrics computed at a probability threshold of 80% and an IoU threshold of 0.30.

**Table 3:** Diagnostic performance of FenoParasite against the reference standard—internal validation (*n* = 159).

Metrics	*A. lumbricoides*	*G. lamblia*
True positive (TP)	25	66
True negative (TN)	134	92
False positive (FP)	0	1
False negative (FN)	0	0
Sensitivity, % (95% CI)	100.0 (86.3–100.0)	100.0 (94.6–100.0)
Specificity, % (95% CI)	100.0 (97.3–100.0)	98.9 (94.2–100.0)
Positive predictive value, % (95% CI)	100.0 (86.3–100.0)	98.5 (92.0–100.0)
Negative predictive value, % (95% CI)	100.0 (97.3–100.0)	100.0 (96.1–100.0)
Accuracy, % (95% CI)	100.0 (97.7–100.0)	99.4 (96.5–100.0)
LR+ (95% CI)	No estimable *	93.0 (13.2–653.3)
AUC-ROC (95% CI)	1.00 (1.00–1.00)	0.98 (0.96–0.99)
Cohen’s κ (AI vs. GS)	1.00	0.99 (0.96–1.00)

95% CI of proportions calculated by the exact Clopper–Pearson method; 95% CI of the AUC by bootstrap (5000 resamples). * Not estimable due to 100% specificity (false positive denominator equal to zero). LR+: positive likelihood ratio; AI: artificial intelligence; GS: gold standard.

**Table 4:** Diagnostic performance of FenoParasite against the reference standard—prospective pilot validation (*n* = 31).

Metrics	*A. lumbricoides*	*G. lamblia*
True positive (TP)	4	13
True negative (TN)	27	18
False positive (FP)	0	0
False negative (FN)	0	0
Sensitivity, % (95% CI)	100.0 (39.8–100.0)	100.0 (75.3–100.0)
Specificity, % (95% CI)	100.0 (87.2–100.0)	100.0 (81.5–100.0)
Positive predictive value, % (95% CI)	100.0 (39.8–100.0)	100.0 (75.3–100.0)
Negative predictive value, % (95% CI)	100.0 (87.2–100.0)	100.0 (81.5–100.0)
Accuracy, % (95% CI)	100.0 (88.8–100.0)	100.0 (88.8–100.0)
AUC-ROC	1.00	1.00
Cohen’s κ (AI vs. GS)	1.00	1.00

95% CI of proportions by the exact Clopper–Pearson method; AI: artificial intelligence; GS: gold standard.

**Table 5:** Interobserver agreement (Reader 1 vs. Reader 2) measured with Cohen’s κ index.

Parasite Stage	Internal Validation, κ (95% CI)	Prospective Pilot Validation, κ (95% CI)
*A. lumbricoides * (egg)	1.00	0.84 (0.53–1.00)
*G. lamblia * (cyst)	0.88 (0.81–0.96)	1.00
*G. lamblia * (trophozoite)	0.95 (0.86–1.00)	0.65 †
Discrepancies resolved by third reader, *n* (%)	25 (15.7)	1 (3.2)

† Wide confidence interval due to the small number of positive events in the pilot phase.

## Data Availability

The anonymized specimen-level analysis dataset supporting all tables and figures in this article, comprising the reference-standard results, the FenoParasite classifications and confidence scores, and the technical image metadata, is openly available in Figshare at doi:10.6084/m9.figshare.33052568. No directly or indirectly identifying patient information is included in the deposited file. Requests for material that is not publicly deposited, including the expert annotation files, the microscopic image corpus, and the trained-model configuration, should be addressed to the corresponding author and will be considered subject to approval by the Research Ethics Committee of the Faculty of Health Sciences, Universidad Privada de Tacna.

## Supplementary Materials

The following supporting information can be downloaded at: https://www.mdpi.com/article/10.3390/tropicalmed11090246/s1, File S1: Completed STARD 2015 checklist; File S2: Completed CLAIM checklist.
