# Supplementary material for: Development and Validation of a Computer Vision-Based Artificial Intelligence System (FenoParasite) for the Microscopic Detection of Ascaris lumbricoides and Giardia lamblia in Stool Samples
Source: Trop Med Infect Dis. 2026 Aug 31;11(9):246. doi: 10.3390/tropicalmed11090246 (PMC13611047; doi:10.3390/tropicalmed11090246)
Supplement: Supplementary file 1 [file tropicalmed-11-00246-s001.zip › File_S2_CLAIM_2024_Checklist_FenoParasite.pdf]

Checklist for Artificial Intelligence in Medical Imaging (CLAIM): 2024 Update

Completed for manuscript: Development and Validation of a Computer Vision-Based Artificial Intelligence System (FenoParasite) for the Microscopic Detection of Ascaris lumbricoides and Giardia lamblia in Stool Samples

Section / Topic	No.	Item	Page / Line	No	NA
**TITLE / ABSTRACT**					
	**1**	Identification as a study of AI methodology, specifying the category of technology used (e.g., deep learning)	pp. 1–2 (title/abstract: computer-vision AI; object detection)		
**ABSTRACT**					
	**2**	Summary of study design, methods, results, and conclusions	p. 2 (Abstract)		
**INTRODUCTION**					
	**3**	Scientific and/or clinical background, including the intended use and role of the AI approach	p. 3; pp. 17–19		
	**4**	Study aims, objectives, and hypotheses	p. 3 (study objective)		
**METHODS**					
Study Design	**5**	Prospective or retrospective study	pp. 4, 8 (§2.1; prospective pilot phase)		
	**6**	Study goal	pp. 3–4 (§1; §2.1)		
Data	**7**	Data sources	pp. 4–5 (§§2.2–2.6)		
	**8**	Inclusion and exclusion criteria	p. 4 (§2.3)		
	**9**	Data pre-processing	Pre-processing steps beyond acquisition/augmentation are not explicitly reported.	X	
	**10**	Selection of data subsets	p. 5 (§2.5; systematic live-field sweep)		
	**11**	De-identification methods	pp. 8, 20 state anonymization, but de-identification methods are not described.	X	
	**12**	How missing data were handled	p. 6 (§ 2.10)		
	**13**	Image acquisition protocol	p. 5 (§2.5)		

Section / Topic	No.	Item	Page / Line	No	NA
Reference Standard	**14**	Definition of method(s) used to obtain reference standard	pp. 2, 4, 13–14 (expert consensus microscopy)		
	**15**	Rationale for choosing the reference standard	pp. 7, 18 (rationale/limitations of expert microscopy)		
	**16**	Source of reference standard annotations	pp. 2, 4, 13–14 (two expert readers; third reader for discrepancies)		
	**17**	Annotation of test set	pp. 4–5, 13–14 (reference microscopy/consensus procedure)		
	**18**	Measures of inter- and intra-rater variability of features described by the annotators	pp. 7, 13–14 report inter-reader κ; intra-rater variability was not assessed.		
Data Partitions	**19**	How data were assigned to partitions	p. 5 (§2.8) describes automated hold-out, but partition proportions/counts could not be reconstructed.	X	
	**20**	Level at which partitions are disjoint	p. 5 (§2.8; image-level split; 159 specimen-level validation samples independent)		
Testing Data	**21**	Intended sample size	p. 3 (§ 2.3)		
**Section / Topic**	No.	Item	Page / Line	No	NA

Section / Topic	No.	Item	Page / Line	No	NA
Model	**22**	Detailed description of model	p. 5 describes Azure Custom Vision CNN, but proprietary architecture is not specified in reconstructable detail.	X	
	**23**	Software libraries, frameworks, and packages	pp. 5, 7 (Azure Custom Vision; Labscope; Excel; Stata 17)		
	**24**	Initialization of model parameters	Not applicable/available to authors: parameter initialization is managed by the proprietary Azure platform.		X
Training	**25**	Details of training approach	p. 5 reports Advanced Training and augmentation, but detailed hyperparameters are not exposed/reported.	X	
	**26**	Method of selecting the final model	Method for selecting the final model iteration is not explicitly reported.	X	
	**27**	Ensembling techniques	Not applicable — no ensemble model was used.		X
Evaluation	**28**	Metrics of model performance	pp. 7, 9–13 (§2.11; §§3.2–3.4)		
	**29**	Statistical measures of significance and uncertainty	pp. 7, 10–13 (95% CIs; bootstrap AUC; Stata 17)		
	**30**	Robustness or sensitivity analysis	No formal robustness/sensitivity analysis is reported.	X	

Tejani AS, Klontzas ME, Gatti AA, et al. Checklist for Artificial Intelligence in Medical Imaging (CLAIM):2024 Update. Radiol Artif Intell 2024;6(4):e240300. https://doi.org/10.1148/ryai.240300

Section / Topic	No.	Item	Page / Line	No	NA
	**31**	Methods for explainability or interpretability	Not applicable — no explainability/interpretability method was applied.		X
	**32**	Evaluation on internal data	pp. 5, 9–11 (§2.8; §§3.2–3.3)		
	**33**	Testing on external data	pp. 4, 12–13, 18 (prospective pilot; lack of true external testing explicitly acknowledged)		
	**34**	Clinical trial registration	Not applicable — this was not a clinical trial.		X
**RESULTS**					
Data	**35**	Numbers of patients or examinations included and excluded	p. 8 reports analyzed sample counts (159 and 31), but screened/excluded counts and a flow diagram are not provided.	X	
	**36**	Demographic and clinical characteristics of cases in each partition	pp. 8–9 report pilot demographics; comparable demographics for the 159-sample internal cohort are not reported.	X	
Model performance	**37**	Performance metrics and measures of statistical uncertainty	pp. 9–13 (§§3.2–3.5; Tables 2–5)		
	**38**	Estimates of diagnostic performance and their precision	pp. 10–13 (diagnostic estimates with 95% CIs; AUC)		
	**39**	Failure analysis of incorrect results	pp. 14–15 (§3.6; confusion matrices)		
**DISCUSSION**					
	**40**	Study limitations	p. 18 (§4.3)		

Section / Topic	No.	Item	Page / Line	No	NA
	**41**	Implications for practice, including intended use and/or clinical role	pp. 17–19 (§§4.3–4.4)		
**OTHER INFORMATION**					
	**42**	Provide a reference to the full study protocol or to additional technical details	A full study protocol or separate technical-protocol reference is not provided.	X	
	**43**	Statement about the availability of software, trained model, and/or data	p. 20 (Data Availability Statement; Figshare and access restrictions)		
	**44**	Sources of funding and other support; role of funders	p. 17		

* Indicate page and/or line number for each checklist item that is present. NA = not applicable.
